# Recruitment of adolescents with suicidal ideation in the emergency department: lessons from a randomized controlled pilot trial of a youth suicide prevention intervention

**DOI:** 10.1186/s12874-020-01117-5

**Published:** 2020-09-14

**Authors:** Matthew Tracey, Yaron Finkelstein, Reva Schachter, Kristin Cleverley, Suneeta Monga, Melanie Barwick, Peter Szatmari, Myla E. Moretti, Andrew Willan, Joanna Henderson, Daphne J. Korczak

**Affiliations:** 1 Department of Psychiatry, Hospital for Sick Children, 555 University Avenue, Toronto, ON M5G 1X8 Canada; 2 Divisions of Paediatric Emergency Medicine and Clinical Pharmacology and Toxicology, Hospital for Sick Children, 525 University Avenue, Toronto, ON M5G 2L3 Canada; 3 Lawrence S. Bloomberg Faculty of Nursing and Department of Psychiatry, University of Toronto, 130-155 College Street, Toronto, ON M5P 1T8 Canada; 4 Department of Psychiatry, University of Toronto, 250 College St, Toronto, ON M5T 1R8 Canada; 5 Research Institute, Hospital for Sick Children, 686 Bay Street, Toronto, ON M5G 0A4 Canada; 6 Clinical Trial Unit, Ontario Child Health Support Unit, Hospital for Sick Children, 555 University Avenue, Toronto, ON M5G 1X8 Canada; 7 Centre for Addiction and Mental Health, 5226-88 Workman Way, Toronto, ON M5J 1H4 Canada

**Keywords:** Clinical trials, Recruitment, Mental health, Suicide, Pediatrics, Youth, Adolescents, Emergency departments, Interventions

## Abstract

**Background:**

Emergency Departments (EDs) are a first point-of-contact for many youth with mental health and suicidality concerns and can serve as an effective recruitment source for randomized controlled trials (RCTs) of mental health interventions. However, recruitment in acute care settings is impeded by several challenges. This pilot RCT of a youth suicide prevention intervention recruited adolescents aged 12 to 17 years presenting to a pediatric hospital ED with suicide related behaviors.

**Methods:**

Recruitment barriers were identified during the initial study recruitment period and included: the time of day of ED presentations, challenges inherent to study presentation, engagement and participation during an acute presentation, challenges approaching and enrolling acutely suicidal patients and families, ED environmental factors, and youth and parental concerns regarding the study. We calculated the average recruitment productivity for published trials of adolescent suicide prevention strategies which included the ED as a recruitment site in order to compare our recruitment productivity.

**Results:**

In response to identified barriers, an enhanced ED-centered recruitment strategy was developed to address low recruitment rate, specifically (i) engaging a wider network of ED and outpatient psychiatry staff (ii) dissemination of study pamphlets across multiple areas of the ED and relevant outpatient clinics. Following implementation of the enhanced recruitment strategy, the pre-post recruitment productivity, a ratio of patients screened to patients randomized, was computed. A total of 120 patients were approached for participation, 89 (74.2%) were screened and 45 (37.5%) were consented for the study from March 2018 to April 2019. The screening to randomization ratio for the study period prior to the introduction of the enhanced recruitment strategies was 3:1, which decreased to 1.8:1 following the implementation of enhanced recruitment strategies. The ratio for the total recruitment period was 2.1:1. This was lower than the average ratio of 3.2:1 for published trials.

**Conclusions:**

EDs are feasible sites for participant recruitment in RCTs examining new interventions for acute mental health problems, including suicidality. Engaging multi-disciplinary ED staff to support recruitment for such studies, proactively addressing anticipated concerns, and creating a robust recruitment pathway that includes approach at outpatient appointments can optimize recruitment.

**Trial registration:**

ClinicalTrials.gov: NCT03488602, retrospectively registered April 4, 2018.

## Background

Mental health problems are common among children and youth. It is estimated that 10 to 20% of Canadians ages 5 to 24 will develop a mental disorder [1]. Youth mental health problems are a source of burden for both the affected child as well as for the family, and can lead to high rates of acute health service use [2]. An early age of onset of mental health problems, in the child and adolescent years, is also associated with increased risk of subsequent suicide-related behaviors (SRBs), which includes suicidal ideation with intent, self-harm with intent, suicide attempt and death by suicide, as well as non-suicidal self-injury [3].

There is a pressing need for evidence-based interventions for reducing SRBs that are effective, feasible and implementable, and that have been tested using rigorous randomized controlled trial (RCT) designs. Many children and youth present to the Emergency Department (ED) seeking care for acute mental health problems, such as SRBs [4–6]. Indeed, EDs are utilized more frequently than outpatient settings by children and youth with acute mental health concerns, including SRB [7]. Moreover, children and youth who present to the ED with SRBs are at increased risk of repeat suicidality and death by suicide, and are at high need of effective interventions [8–12]. As a result, there is a need for well-designed RCTs of potentially effective suicide prevention interventions for youth who present to the ED. [13, 14]

Recruitment productivity, i.e., a trial’s effectiveness in recruiting and randomizing participants, is a vital factor for clinical trial success; many trials suffer from problems of recruitment, regardless of their setting and clinical field. Two United Kingdom studies found that the majority of medical trials reviewed (60 to 69%) failed to meet the originally proposed recruitment targets; neither study indicated whether pediatric trials were included in the analyses such that child- or youth- specific figures were not able to be determined [15, 16].

While recruitment barriers are common to all clinical trials, evidence from a large, international study of both adult and pediatric RCTs suggests that trials conducted in acute (i.e., critical or emergency care) settings, however, are at greater risk for premature discontinuation due to poor recruitment compared with RCTs in non-acute settings [17]. There may be additional recruitment barriers present in the ED compared with other settings [18–20]. There have been proposals that investigators publish enrollment data, as a measure of recruitment productivity, alongside trial findings for the benefit of future trials [21].

Although many recruitment challenges of ED-based RCTs have been identified, we are not aware of any previous research addressing those recruitment challenges in the specific setting of ED-based RCTs of youth mental health interventions. Such trials may incur the same ED recruitment barriers as other medical fields’ trials, but some recruitment barriers may be unique to youth seeking acute mental health care.

We present data from a pediatric hospital ED-based youth suicide prevention RCT, that recruited adolescents aged 12 to 17 years who presented to the ED with SRBs [22]. Participants were randomized to receive a brief outpatient youth- and family-based psychotherapy (YSP) intervention or a telephone-based case navigation support, both in addition to usual care. In the months following study initiation we documented challenges to participant recruitment and explored innovative strategies to refine and enhance recruitment productivity.

## Objectives

The objectives of the current paper were twofold. The primary objective was to explore barriers to participant recruitment in the ED for a pilot RCT of a suicide prevent strategy (the YSP pilot trial), and to compare the initial screening-to-randomization ratio with that following implementation of an enhanced recruitment strategy aimed at addressing identified barriers. Our second objective was to compute a measure of recruitment productivity for published clinical trials of youth suicide prevention interventions among patients with SRBs which included the ED as a site of recruitment.

## Methods

### Examination of potential barriers to ED participant recruitment

The first objective is to examine the potential barriers to ED participant recruitment for a pilot RCT prevention strategy.

#### YSP pilot trial setting

The protocol of the ED-based youth suicide prevention (YSP) trial has been described in detail elsewhere [22]. In brief, the trial measures the effectiveness of a 6-week, manualized youth- and family-centered suicide prevention strategy (SPS) for adolescents ages 12 to 17 who present to the ED with suicide related behaviors. Participants are randomized to the SPS condition, or the control comparator condition which receives case navigation (NAV) on a 1:1 ratio stratified by age and sex. The primary outcome was change on the Suicidal Ideation Questionnaire-Jr [23] at 6-month follow-up. Recruitment was initiated in March of 2018 and continues at the time of publication.

The RCT operates within a large, urban paediatric hospital in Ontario, Canada. Following medical assessment by the ED team, youth who present to the ED with SRB may be discharged by the ED staff or assessed by the psychiatry on-call team, which is comprised of psychiatrists, psychiatry trainees and nurse practitioners. Youth who are deemed to be unsafe for discharge by the psychiatry service are admitted to the psychiatry inpatient unit; patients that are discharged from the ED are provided with recommendations for follow-up, including information regarding community mental health resources. Follow-up recommendations may include referral to the hospital’s Psychiatry Urgent Care Clinic (UCC), which provides one-time psychiatric assessment, and short-term crisis treatment designed to help bridge patients to community resources.

#### YSP pilot trial baseline ED recruitment strategy

**Fig. 1:**
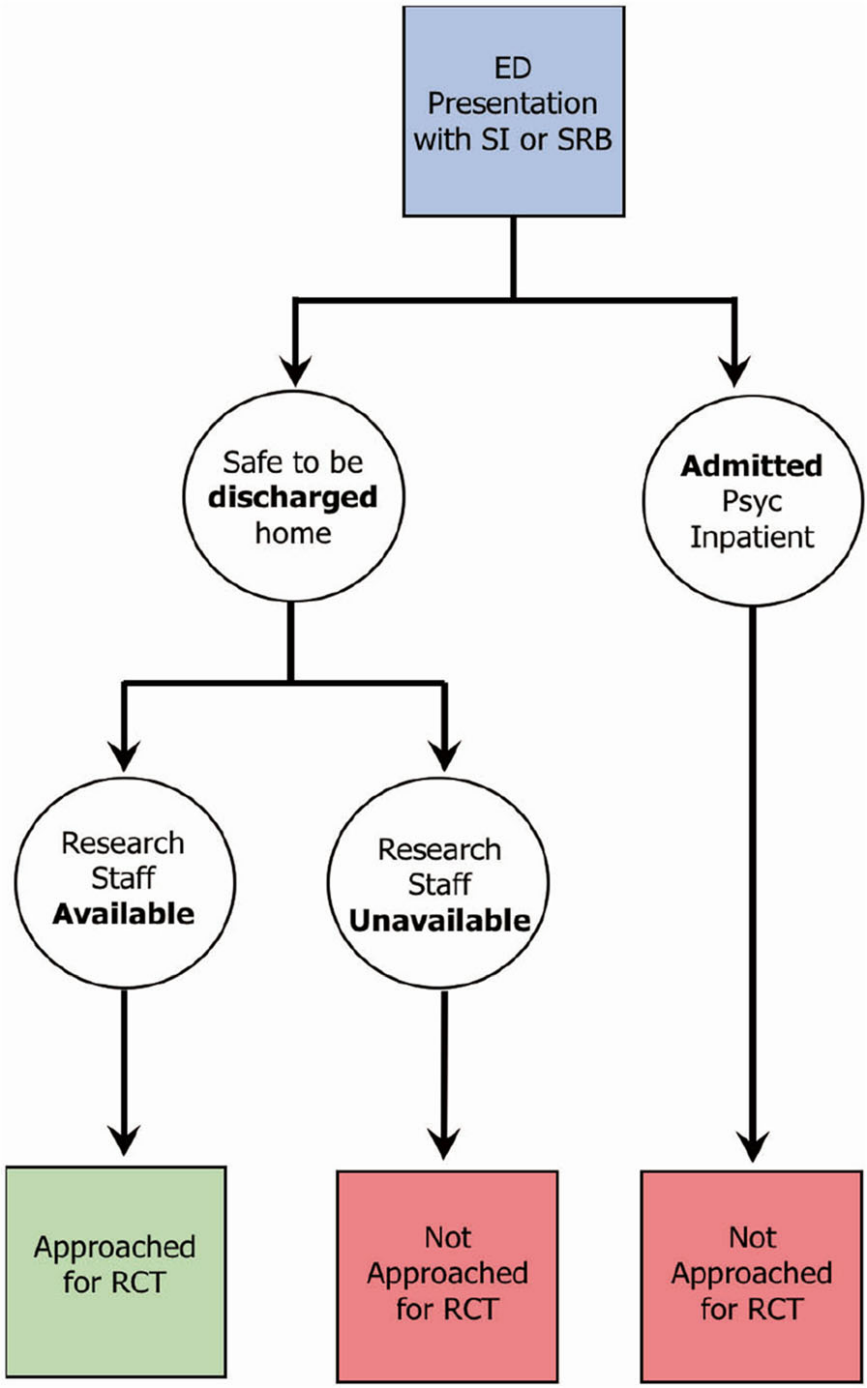
Original RCT Recruitment Pathway

The initial recruitment protocol (See Fig. 1) occurred exclusively within the hospital’s ED and leveraged a preexisting program of research nurses who were responsible for identifying, screening and consenting patients for multiple clinical trials ongoing within the ED. One research nurse was scheduled each day for a 12-h shift starting either at 6 AM or at 8 AM to provide the study with coverage during working hours of a typical day shift. The research nurse monitored the hospital’s electronic patient tracking system for patients who were between the ages of 12 to 17 years and who presented to the ED for the primary reason of SRB; these patients were designated as eligible by pre-screen.

The research nurse approached a clinical member of a pre-screen eligible patient’s circle of care (i.e., physician, psychiatrist, nurse or social worker) and requested that the clinician inquire as to the patient’s and caregiver’s interest in learning about a research study. If the youth and family were interested in the trial, the nurse administered the eligibility screening measures. The original inclusion criteria included (1) ≥ 12 and ≤ 17 year; (2) presenting to the ED with Suicidal Ideation Questionnaire-Jr [23] (SIQ-Jr) score ≥ 31; (3) has a participating parent or caregiver who is able to communicate in English or is willing to communicate using a hospital-organized translator; (4) living in the hospital catchment area; and (5) access to a telephone (mobile or land line). Exclusion criteria included (1) active psychosis or mania (mood elevation score ≥ 3 on the Schedule of Affective Disorders and Schizophrenia for School-Aged Children [24] (KSADS) screen; (2) moderate to severe intellectual disability, and/or autism based on parent report or clinical chart, and (3) admitted to hospital for self-harm and suicide-related behaviors. Hospitalized patients were excluded at the pilot study site as the intervention under investigation has been implemented by the inpatient psychiatry program. Eligible patients were consented immediately after screening. The research nurse then notified the study coordinator to contact the family by phone the following day and book the participant’s baseline study visit.

#### YSP trial baseline recruitment barriers

During the first 2 months of study initiation, only three of the 15 (33%) participants approached in the ED were recruited and randomized into one of the study arms. This was lower than the team’s initial recruitment estimate of 4 participants per month, thereby alerting the study team to the possibility that barriers for recruitment may be present.

Barriers were identified from several sources from the beginning of the study and throughout the recruitment period. Informal feedback on the recruitment process was elicited from research staff (including research nurses and the study coordinator), as well as clinical staff (ED and the UCC) and summarized by the research team. The research staff also completed a review of the chain of events within the recruitment process identified. They identified the following barriers to recruitment: the timing of approach in the ED, staffing models and roles; and youth and family factors.

First, the working hours of the clinical research nurses were not optimal for the daily cycle of ED presentations for SI or SRB. The team observed that many patients with SRB presented to the ED in the evening hours or overnight. This resulted in many potential participants missed as the research nurse shift ended prior to the assessment by the ED team. Also, many youths with SRB who did present to the ED during the research nurse’s hours did so near the end of the shift. As such, by the time the youth was triaged, placed in a room and ready for study eligibility screening, the study recruitment nurses were no longer available to meet with potentially eligible youth.

Second, shift work is common within emergency medicine, such that the study research team would not encounter the same ED staff members over a period of days or weeks. Research staff members found that frequent re-introduction of the study objectives to ED physicians attending to eligible patients was required, increasing the amount of study communication time invested for the research staff. Research staff found that ED physicians, while supportive of the study, were often were too busy or forgot to page the research nurse prior to discharging a potentially eligible patient, thereby missing the opportunity to recruit the patient into the study.

Third, the research nurses and coordinator observed that it could be difficult to approach patients in the ED due to the frequent interruptions from the circle of care members, such as clinical nurses conducting regular assessment of vital signs, or an ED social worker completing an assessment. This may not only have the effect of prolonging the length of the screening, it may have also deterred some participants from engaging in the screening or consent process.

Fourth, adolescents presenting to the ED with SRB and their accompanying family members are acutely distressed and frequently within a state of crisis. Some family members expressed to research staff that they found it difficult to accept their child’s suicidality, or that they believed that their child’s distress was situational and would self-resolve, obviating the need for services, including those that may be provided through study participation.

Finally, on discharge from the ED, adolescents and families were provided with information about potentially helpful community resources and referrals to outpatient mental health clinics were made by the clinical team. Research nurses and staff observed that some families appeared overwhelmed by the information provided, and as such, declined to hear about further opportunities, including suicide prevention intervention research. Some patients and families expressed interest in the study and requested to be contacted at a later date, after they had reviewed all the resources provided by the ED, posing the risk of loss of potentially eligible patients for recruitment.

### YSP pilot trial recruitment productivity

The second objective was to measure the recruitment published trials of youth suicide prevention interventions and to compare it with the productivity of the YSP pilot trial.

In order to determine the recruitment productivity in the published literature, a recent systematic review and meta-analysis was used to identify published trials of adolescent and youth suicide prevention interventions was used [25]. Full-text review of studies included in the meta-analysis was undertaken by the research staff and recruitment data were extracted from the original publications. Variables included (1) the recruitment setting, (outpatient or ED-based); (2) the total number of patients screened for participation; (3) the number of patients eligible; and (4) the number of patients randomized. To assess the difference in recruitment productivity, a randomization-to-screening ratio was calculated. Previous research has reported the ratio between participants enrolled to the number of individuals screened (screening-to-enrollment) as a measure of recruitment productivity [21]. However, as patients may be enrolled several days prior to baseline assessment and randomization, enrollment alone does not sufficiently capture recruitment efficiency. Patients may enroll in the trial, and then withdrawal before randomization. Therefore, the screening-to-randomization ratio was used for this analysis.

As published adolescent and youth suicide prevention trials provided only the number of screening and randomizations over the course of the total recruitment period, a single, total ratio was produced for each study. Published reports were not included if a ratio could not be calculated, e.g., the authors did not report the number of screens that were conducted. Two mean screening-to-randomization ratios were produced: one for studies that included the ED as a recruitment site in addition to any other recruitment site (e.g., inpatient or outpatient settings) and one for those that recruited in outpatient settings only.

In order to calculate recruitment productivity for the YSP trial, the number of patients screened, i.e., those that completed the eligibility questionnaires, and the number of consented patients randomized, i.e., assigned to either the YSP or control condition, was collected as part of a master log.

A total ratio for the YSP was calculated. In addition, a ratio for the first four months of the recruitment period (March–June 2018), which reflects the period of time in which the original protocol was in effect, and compared with the ratio of the same four months of the following year (March–June 2019) in order to compare changes in recruitment productivity. A year-over-year comparison was used in order to control for seasonal variations in the number of pediatric ED presentations for SI or SRB [26]. R statistical software was used for all statistical analysis [27].

## Results

### Solutions to YSP trial recruitment barriers

After observing the recruitment process in the first two months of the trial, the research team concluded that the timing of approach was the most significant recruitment barrier. Many eligible patients were being missed, as they were discharged from the ED home prior to contact with the research team member for assessment of study eligibility.

**Fig. 2:**
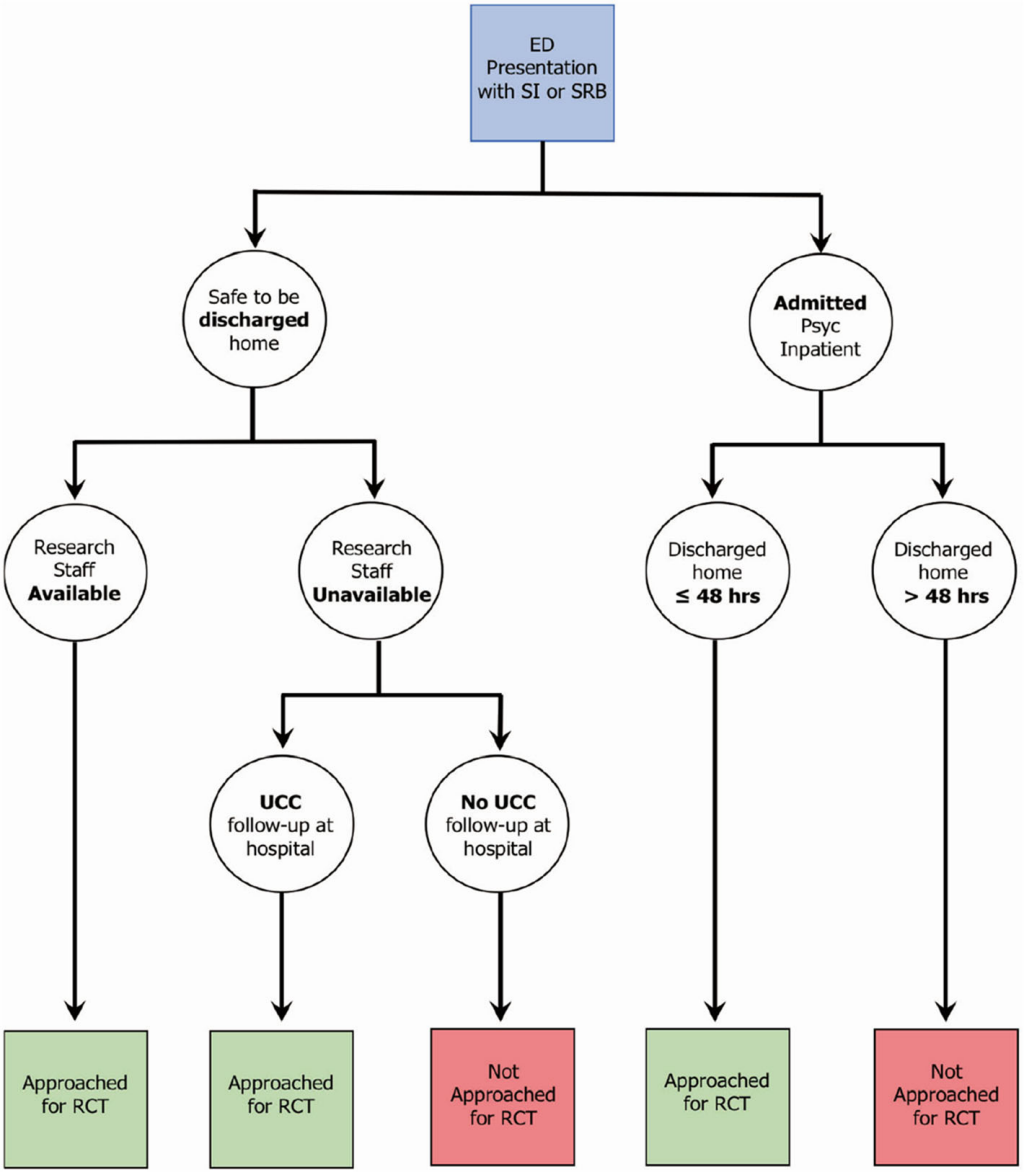
Amended RCT Recruitment Pathway

The study investigators and study coordinator addressed this barrier by developing a more flexible recruitment pathway (see Fig. 2). As the study intervention was not administered within the ED, it was possible to conduct the eligibility screen after the patient had been discharged home. The research team was able to implement a process whereby baseline and randomization occurred within two weeks of the index presentation to the ED without incurring any significant change to the study timeline.

It was further observed by the research team that a large proportion of potentially eligible adolescents were referred by the ED clinical team to the hospital’s psychiatry outpatient urgent care clinic (UCC). The protocol was subsequently amended in order to approach these patients either within the ED or at the time of their UCC clinic appointment. Engagement of the UCC staff facilitated contact with youth and families following discharge. The study research coordinator began to attend morning UCC rounds where ED-referred patients were triaged for this purpose.

n addition, the research team amended the study protocol to include patients who were briefly admitted to the psychiatric inpatient unit for up to 48 h. All hospitalized patients were initially excluded in order to avoid the effects of potential co-interventions (administered during admission) on study participant outcomes. However, the inpatient team confirmed that patients who remained in hospital for less than 48 h engaged in limited inpatient programming, and thus, could be eligible for study inclusion. These amendments enabled the study team to approach ED-referred patients to UCC at the time of their outpatient UCC appointment or approach patients who had been briefly hospitalized at the time of discharge. These changes addressed several important recruitment barriers:

First, as a result of the amendment to the recruitment pathway the challenge of out-of-working hours ED presentation was addressed, as the majority of patients who presented to the ED for SRB on evenings and weekends could be approached at the time of contact with the UCC.

Second, the study moved from a recruitment pathway that was staffed exclusively by ED research nurses, who were participating in several ED-based studies in parallel, to one in which study research assistants (RAs) were dedicated to the recruitment of suicide prevention study participants in either the UCC or ED setting. RAs were able to identify full-time clinical team members in the ED and the UCC who were aware of the study eligibility criteria and who could act as study champions by actively engaging in the effort to recruit within their clinical setting, and by alerting study staff when potential candidates presented.

The RCT team also increased study awareness of the ED clinical staff by presenting the study in several forums, including ED staff meetings and monthly ED research council meetings. Presentations at meetings for ED-covering psychiatrists, relevant social workers, and resident trainees in psychiatry on-call to the ED further increased study awareness. As a reminder, the study inclusion/exclusion criteria were incorporated into the handover communication that the psychiatry on-call team received at the start of each of their on-call shift. The research team also designed a short information flyer regarding the study and the basic eligibility criteria and made it available to all staff. Finally, the principal investigators and research staff identified champions motivated to recruit for the study among the core ED staff, including physicians, social workers and nurses.

Third, eligibility screens and consent procedures during outpatient UCC visits could be conducted without interruption from clinical staff. This meant that the length of the procedures was more consistent, and that the family could attend to the screening without interruption. When screenings completed within the ED were delayed due to interruptions, the study RAs could coordinate with the patient to complete the screening at a different time after discharge.

Finally, as staff psychiatrists and trainees within the outpatient clinic became familiar with the study, they increasingly incorporated information about the study during the wrap-up of their clinical encounter. Integration of the study into the UCC clinic appointment may also have increased the patient and family understanding of the study context and receptivity to participating in a research study. The research staff observed that, following the UCC appointment, adolescents and families appeared to have a greater appreciation of the severity of the adolescent’s condition, and a more accurate understanding of the resources that may be available to them.

### Recruitment productivity for published RCTs and the YSP pilot trial

The screening-to-randomization ratio was calculated for published trials of youth suicide prevention interventions that included the ED as a site of recruitment. Twenty-one trials included in the published systematic review contained sufficient recruitment information to calculate the screening-to-randomization ratio (Table 1). The overall ratio for RCTs that included ED recruitment (*n* = 14) was 3.2:1 (range: 1–10.7:1), and 70% of patients screened were eligible. In comparison, the overall ratio for trials that recruited exclusively in outpatient settings (*n* = 7) was 1.5:1 (range: 1–2.7:1), and 85.4% of patients screened were eligible.

**Table 1:** Recruitment data for published suicide prevention RCTs for youth [25]

Study (Year)	Recruitment Includes ED (Y/N)	Screened	Eligible	Randomized	Screen/Randomized
Asarnow et al. (2017) [28]	Y	140	60	42	3.3
Bertolote et al. (2010) [29]	Y	2973	2896	1867	1.6
Diamond et al. (2010) [30]	Y	341	129	66	5.2
Husain et al. (2014) [31]	Y	250	236	221	1.1
King et al. (2006) [32]	Y	1316	986	289	4.6
King et al. (2009) [33]	Y	2493	1050	448	5.7
King et al. (2015) [34]	Y	526	49	49	10.7
Mehlum (2016) [35]	Y	294	189	77	3.8
Ougrin et al. (2011) [36]	Y	96	78	70	1.4
Pineda & Dadds (2013) [37]	Y	64	64	48	1.3
Rossouw & Fonagy (2012) [38]	Y	120	110	80	1.5
Rudd et al. (1996) [39]	Y	303	302	302	1.0
Spirito et al. (2002) [40]	Y	82	82	76	1.0
Wharff et al. (2019) [41]	Y	330	287	142	2.3
Mean screening-to-randomization ratio for ED- recruitment RCTs:					**3.2**
Carter et al. (2010) [42]	N	112	96	76	1.0
Cooney et al. (2010) [43]	N	35	33	29	1.2
Green et al. (2011) [44]	N	402	394	366	1.1
Harrington et al. (1998) [45]	N	435	288	162	2.7
Hazell et al. (2009) [46]	N	138	133	72	1.9
Slee et al. (2008) [47]	N	100	92	90	1.1
Wood et al. (2001) [48]	N	83	79	63	1.3
Mean screening-to-randomization ratio for outpatient-recruitment RCTs					**1.5**

In the first 18 months of YSP Pilot recruitment, 148 ED patients were pre-screened as eligible for the study. Of these, 88 were screened, 65 (73.8%) were eligible, 51 (57.9%) were consented and 45 (51.1%) were randomized, yielding a screening-to-randomization ratio of 1.9:1 for the total recruitment period. The screening-to-randomization ratio for the study was 2:1 (SD = 1.3, range: 0.7–5), which is at the lower bounds of screening-to-randomization ratios of previously published adolescent RCTs in the field.

The screening-to-randomization ratio for the first 4 months of study recruitment under the original recruitment protocol was 3:1. Following implementation of the enhanced recruitment strategy, the screening-to-randomization ratio for the same 4-month period during the next year decreased significantly to 1.8:1.

## Discussion

Recruitment productivity is integral to the success of RCTs for new and potentially effective treatments, including youth mental health interventions. In this study, we found that the screening-to-randomization ratio decreased, indicating more successful recruitment (ie fewer youth screened for every participant randomized) for the YSP pilot RCT under the enhanced recruitment protocol compared with the baseline recruitment protocol. This ratio is among the most successful recruitment rates in the reported literature within ED settings (ranges 1 to 10.7) [25].

Previous literature has demonstrated that the recruitment of ED populations has yielded lower recruitment rates than RCTs conducted in non-ED settings [15–20]. It is, therefore, necessary for investigators to assess recruitment productivity along a study continuum, and to compare these results with those of trials in similar populations and settings. One systematic review of studies of adult patient attitudes towards research in the ED noted that the consent process may be more challenging in the ED compared with other settings due to the time-sensitive nature of some emergent conditions. [19] Patients may feel that the process is rushed, or may not be receptive or attentive to hearing about research because of their acute state and the anxiety which accompanies it. Investigators have also identified several general recruitment challenges that are particularly salient for ED-based RCTs, including: protocols for identifying and screening patients; the timeframe for consenting and enrolling patients; and study retention post ED discharge [20].

The initial recruitment rate in the current study incurred similar challenges, including a scheduling mismatch between staffing and the timing of patient presentation in the ED, and environmental conditions in the ED (e.g., lack of privacy or quiet environment for study screening and consent). The study team addressed these barriers by assessing the clinical pathway for the target population and modifying the protocol in order to approach patients missed in the ED at outpatient appointments. We also increased staff awareness of the study by producing and circulating information for clinical staff in both the ED and outpatient settings. Engagement of study champions in all clinical settings required significant, targeted, and ongoing communication efforts which are time-consuming yet crucial to improving study recruitment rates.

Our findings highlight the importance of patient and family engagement in the design and implementation of clinical trials [49]. The feedback that we received from youth and families provided us with valuable insights for optimizing recruitment and identifying barriers for youth experiencing mental health crises. Engaging with patients and families prior to study initiation may be of even further value in identifying and addressing potential barriers before they arise.

Finally, we used our recruitment data as a means of evaluating the success of our amended recruitment pathway, which allowed ongoing modifications and flexibility based on actual experience and lessons learned by various team members on-the-go. Our findings suggest that when possible, trial investigators should implement improvements to recruitment protocols by first carefully assessing the study recruitment environment and leveraging relationships with clinicians who are able to assist in the recruitment process.

There are limitations to our analysis. First, changes in recruitment productivity may have resulted from factors unrelated to our amended recruitment pathway, e.g., an increase in the clinical staff’s familiarity with the study could have increased recruitment. Second, the screening-to-randomization ratio is one of several different methods for measuring recruitment productivity (e.g., screening-to-enrollment ratio) and other measures were not explored. However, we argue that screening (i.e., first contact with the study protocol) and randomization (i.e., exposure to one of the two study arms) are the most meaningful points across which to examine recruitment productivity along the study recruitment pathway.

## Conclusions

Recruitment enhancing processes can be highly successful in improving the youth RCT recruitment productivity in an acute ED setting. Indicators of recruitment productivity are useful metrics that can provide RCTs, particularly pilot RCTs, with an assessment of success, determine whether recruitment enhancement efforts are required, and help identify challenges to be addressed. These indicators may also be an effective means by which to monitor progress and success over the course of the study. Trial investigators should consider including recruitment meta-data, such as the monthly screening-to-randomization ratio, in publications of study results to assist investigators in the design of successful recruitment pathways and enhancers in future research studies.

## Data Availability

Primary data analysed during the current study are not publicly available as data contain potentially identifying and sensitive information. Secondary data analysed for the current study are available in the published literature and referenced as such throughout the manuscript.

## Abbreviations

ED: Emergency Department
NAV: Case Navigation
RCT: Randomized Controlled Trial
SI: Suicide Ideation
SRB: Suicide Risk Behaviour
UCC: Urgent Care Clinic
YSP: Youth Suicide Prevention
